# Clinical Observation of a Novel Moisturizing Cream for Reducing Neurovascular Hyper‐Reactivity in Sensitive Skin

**DOI:** 10.1111/jocd.70287

**Published:** 2025-07-30

**Authors:** Li Jiang, Jing Ouyang, Dian Wu, Miao Lai, Shuangli Yu, Zeheng Xu, Li He

**Affiliations:** ^1^ Department of Dermatology First Affiliated Hospital of Kunming Medical University Kunming China; ^2^ Skin Health Research Center, Yunnan Characteristic Plant Extraction Laboratory Kunming China

**Keywords:** functional skincare products, neurovascular hyper‐reactivity, sensitive skin

## Abstract

**Background:**

Sensitive skin is a syndrome characterized by unpleasant sensations in response to external stimuli. Its pathogenesis is linked to impaired skin barrier function, leading to neurovascular hyper‐reactivity and inflammation. While topical functional skincare products are known to repair sensitive skin, there are limited reports on products specifically targeting the reduction of neurovascular hyper‐reactivity.

**Objective:**

To evaluate the efficacy and safety of a novel cream in improving neurovascular hyper‐reactivity and subjective symptoms in sensitive skin.

**Methods:**

This randomized, double‐blind, self‐controlled trial included 35 participants. One side of the face was treated with experimental cream (Group A) and the other with control cream (Group B), applied twice daily for 28 days. Follow‐up assessments were performed on Day 0, Day 7, Day 14, and Day 28, with 28‐day post‐trial safety monitoring. Evaluations included clinical evaluation by physician, participant self‐assessment of symptoms, measurements of skin physiological parameters and current perception threshold (CPT), lactic acid stinging test (LAST), capsaicin pain test (CAT), product usage experience, and participant tolerance.

**Results:**

The experimental cream demonstrates good safety and user experience. Both the experimental and control creams effectively improve symptoms and signs of sensitive skin, increase SC hydration, and lower TWEL, LAST, and DLQI scores. Additionally, the experimental cream outperforms the control cream in improving pruritus, erythema, and TWEL. The experimental cream also has the unique advantage of reducing CAT scores and increasing CPT.

**Conclusion:**

The new cream has good safety and can effectively reduce neurovascular hyper‐reactivity, alleviating symptoms in individuals with sensitive skin.

## 1 Introduction

Sensitive skin is internationally defined as a syndrome in which the skin experiences unpleasant sensations (including tingling, burning, pruritus, pain, or stinging) in response to various mild stimuli [1]. These stimuli include various factors such as chemical, physical, and psychological triggers, with the most significant being cosmetics. Following cosmetics, other contributing factors are wet air, air conditioning, and heat [2]. Sensitive skin can occur on any part of the body, with a particular predilection for the face. Its global prevalence can be as high as 71% among adults [3].

Barrier dysfunction is the underlying cause of sensitive skin. It affects not only the traditional “brick‐and‐mortar” structure of the skin but also the stratum granulosum and stratum spinosum [4, 5]. Once the epidermal permeability barrier is compromised, various endogenous and exogenous stimuli, such as heat, UV radiation, and substance P, are more likely to penetrate the skin, activating the transient receptor potential vanilloid 1 (TRPV‐1). Activated TRPV‐1 triggers neurogenic inflammation [6], skin immune responses [7], and vascular hyper‐reactivity [8, 9], leading to symptoms like pain, pruritus, and burning [10, 11, 12].

Individuals with sensitive skin often experience neurogenic symptoms such as pruritus, burning, tightness, and tingling, along with signs like facial flushing and erythema. These issues significantly impact both their physiological and psychological well‐being, making sensitive skin a growing global concern. Currently, treatment strategies for sensitive skin, in addition to pharmacological and physical therapies, increasingly rely on topical skincare products with soothing and moisturizing effects as an important adjunct to sensitive skin management. Based on the pathogenesis of sensitive skin, previous skincare products have predominantly focused on skin barrier repair. For instance, our team's prior research has shown that a moisturizing cream containing *prinsepia utilis oil* effectively repairs the skin barrier of sensitive skin; however, they lack active ingredients that reduce neuro‐hyper‐reactivity [13]. With the deepening understanding of the pathogenesis of sensitive skin, the role of neurovascular hyper‐reactivity has become increasingly critical. Moreover, various neuropathic symptoms require immediate attention. Therefore, it is necessary to develop a skincare product that, in addition to repairing the skin barrier and possessing anti‐inflammatory properties, can also reduce neurovascular hyper‐reactivity and effectively alleviate subjective symptoms.

This study uses Winona Sensitive Moisturizing Special Care Cream (First Generation) as the control cream. The experimental cream, based on the composition of the control cream, includes four additional active ingredients: prinsepia utilis royle polysaccharides, *physalis alkekengi* calyx, *salvia miltiorrhiza* root, and *porphyridium cruentum* extract. Prinsepia utilis Royle polysaccharides [14] can promote the expression of Claudin family proteins, thereby enhancing the skin barrier, and also inhibit the content of substance P to reduce neuro‐hypersensitivity and inflammation. *Porphyridium cruentum* [15] can activate endothelin‐1 to increase the elasticity of skin blood vessels, with anti‐inflammatory and moisturizing effects. Salvia root extract reduces the expression of TRPV1 and lowers levels of inflammatory cytokines IL‐1β, IL‐6, and TNF‐α [16], thereby reducing neurovascular hyper‐reactivity. *Physalis alkekengi* calyx contains various compounds that primarily suppress inflammatory responses [17]. Other ingredients such as purslane, sea buckthorn oil, sodium hyaluronate, and β‐glucan work through various pathways to repair the skin barrier, inhibit inflammation, and moisturize. This study aims to evaluate the effectiveness and safety of the experimental cream in improving neurovascular hyper‐reactivity and symptoms of sensitive skin through clinical observation.

## 2 Materials and Methods

### 2.1 Study Design

This is a randomized, double‐blind, self‐controlled clinical study conducted in the Dermatology Department of the First Affiliated Hospital of Kunming Medical University, Yunnan, China. The study protocol has been successfully registered with the Ethics Committee of Kunming Medical University ((2024) Ethics L No. 43) and has received ethical review and approval. Before enrollment, all participants were informed about the potential benefits and risks of the study and signed an informed consent form.

### 2.2 Study Subjects

A total of 35 participants were included in this study. The inclusion criteria were as follows: (a) aged 18–65 years, regardless of gender; (b) identified as having sensitive skin (score > 18) based on the Huaxi sensitive skin questionnaire; (c) both primary and secondary sensitive skin conditions were included; (d) participants must have a reasonable expectation to comply with all evaluation requirements, use the products according to the study protocol, and complete skin testing and follow‐up visits, without altering their usual diet or undergoing facial cosmetic or aesthetic surgeries during the study; (e) voluntary participation with signed informed consent. The exclusion criteria were: (a) severe or progressive diseases (e.g., cancer, uncontrolled diabetes, epilepsy, etc.); (b) known allergies to any ingredient in the study products; (c) pregnancy, planned pregnancy during the study, or breastfeeding; (d)use of prohibited medications (e.g., corticosteroids, immunosuppressants, antihistamines, photosensitive drugs) within 14 days prior to the study; (e) other medical or professional reasons that may affect the study outcomes as determined by the investigators.

### 2.3 Study Products

The test product (Cream A) is Winona Sensitive Moisturizing Special Care Cream (2nd Generation). The control product (Cream B) is Winona Sensitive Moisturizing Special Care Cream (1st Generation). Both creams were manufactured and supplied by Beitaini Biotechnological Co. Ltd. The composition of creams A and B is shown in Table 1.

**TABLE 1:** The Compositions of Cream A and Cream B.

		Compositions of cream		
Cream A	Aqua, glycerin, pentylene glycol, *butyrospermum Parkii* (shea butter), tridecyl, trimellitate, dimethicone, glyceryl stearate, prinsepia utilis oil, sodium hyaluronate, *portulaca oleracea* extract, beta‐glucan, acrylates/c10‐30 alkyl acrylate crosspolymer, xanthan gum, butylene glycol, aminomethyl propanol			
Cream B	Aqua, glycerin, pentylene glycol, butylene glycol, *butyrospermum Parkii* (shea butter), *sclerocarya birrea* oil, trimellitate, dimethicone, acrylates/c10‐30 alkyl acrylate crosspolymer, batyl alcohol, lecithin, P‐hydroxyketone, sodium hyaluronate, aminomethyl propanol, prinsepia utilis oil, *portulaca oleracea* extract, *physalis alkekengi* calyx extract, prinsepia utilis Royle polysaccharides, *salvia miltiorrhiza* root extract, *porphyridium cruentum* extract, beta‐glucan, tocopherol, hydroxypropyl cyclodextrin			

### 2.4 Treatments

After cleansing their face with water in the morning and evening, participants were instructed to apply two pumps of either Cream A or Cream B to each side of their face, spreading it evenly and gently massaging until absorbed. During the trial, one side of the face always used Cream A (Group A), while the other side used Cream B (Group A). At the same time, no other products, such as cosmetics or other skincare products, were applied to the face during the trial. The investigator verified product usage and checked the remaining amount in the containers at each follow‐up visit to ensure correct application and dosage for each participant. Follow‐up evaluations were conducted on Day 0, Day 7, Day 14, and Day 28, with safety monitoring performed within 28 days after the study concluded.

### 2.5 Assessment Methods

#### 2.5.1 Participant Symptom Self‐Assessment

The participants sequentially rate four symptoms, including tightness, pruritus, burn, and sting, according to the following scale: 0 = none, 1 = mild, 2 = moderate, 3 = severe.

#### 2.5.2 Clinical Evaluation by Physician

The physician sequentially scores the four signs of erythema, dryness, roughness, and desquamation in the participants according to the following scale:0 = none, 1 = mild, 2 = moderate, 3 = severe, and 4 = very severe.

#### 2.5.3 Dermatology Life Quality Index (DLQI) Questionnaire

The questionnaire was used to evaluate the participants' recent quality of life. It consists of 10 questions, and the total score is obtained by summing the scores of all the questions, with a maximum possible score of 30.

#### 2.5.4 Facial Imaging With VISIA‐7 (CANFIELD, USA)

Facial images were taken of the participants' front, left, and right sides, with consistent lighting parameters and positioning. Key analysis parameters included erythema area, L* value, and a* value. The software, utilizing image segmentation and color space principles, calculates the erythema area: the erythema regions on the face are measured using a rectangular frame, and the erythema area is then segmented out. The ratio of the number of pixels in the erythema region to the total number of pixels in the rectangular frame represents the erythema area ratio. Additionally, L* value and a* value were analyzed using IPP software.

#### 2.5.5 Skin Physiological Measurements

**Stratum corneum hydration (SC hydration)**: Measured with a Corneometer CM825 (Courage&Khazaka, Germany) at the highest point of the cheekbone. Measurements were taken three times, and the average value was calculated.

**Trans‐epidermal water loss (TEWL)**: Measured using a Tewameter TM300 (Courage&Khazaka, Germany) at the highest point of the cheekbone. Measurements were taken three times, and the average value was calculated.

**Erythema index (EI)**: Measured using a Mexameter MX18 (Courage&Khazaka, Germany) at the highest point of the cheekbone. Measurements were taken three times, and the average value was calculated.

**Sebum**: Measured using a Sebumeter SM815 (Courage&Khazaka, Germany) on the forehead, 2.5 h after cleansing, with one measurement.

#### 2.5.6 Current Perception Threshold (CPT)

Measured using a Neurometer@CPT (Neurotron, USA) at a point 2 cm lateral to the nasolabial fold. Three measurements were taken, and the average value was calculated. The CPT was assessed at a frequency of 5 Hz to evaluate C‐fiber nerve function (pain sensation, temperature, and slow pain), reflecting skin nerve sensitivity [18].

#### 2.5.7 Lactic Acid Stinging Test (LAST) and Capsaicin Test (CAT)

Evaluated at Day 0 and Day 28. The LAST involved applying a 10% lactic acid solution to the nasolabial fold and assessing discomfort (itching, stinging, or burning) at 2.5 and 5 min. The CAT was performed after the lactic acid discomfort subsided, applying 0.1‰ capsaicin to a point 1 cm outside the nasolabial fold.

#### 2.5.8 Product Usage Experience

Participants rated their experience with the two products during the 28‐day visit. The subject rates the product usage experience based on the following criteria (Table 2).

**TABLE 2:** Product usage experience rating scale.

Item	Product usage experience			
	Strongly disagree	Somewhat disagree	Somewhat agree	Strongly agree
No oily residue left on the skin	1	2	3	4
Rapid absorption	1	2	3	4
Improved skin comfort	1	2	3	4
Visible skin repair	1	2	3	4
Mild and non‐irritating	1	2	3	4
Effective improvement in skin roughness	1	2	3	4
Effective soothing of skin redness	1	2	3	4
Improvement in skin tightness	1	2	3	4
Improvement in skin itchiness	1	2	3	4
Effective relief of skin desquamation	1	2	3	4

#### 2.5.9 Tolerability Assessment and Adverse Reaction Recording

On Days 7, 14, and 28, the investigator assessed product tolerance based on the following scale: 0 (Excellent): No discomfort or symptoms. 1 (Good): Minor, transient discomfort, no discontinuation required. 2 (Fair): Persistent discomfort or symptoms, no discontinuation required. 3 (Poor): Discomfort and/or symptoms requiring discontinuation. Adverse reaction record: On Days 7, 14, 28, and 28 days post‐study, participants were asked about adverse reactions (e.g., pruritus, erythema, papules) and any events were recorded.

### 2.6 Statistical Analysis

Statistical analysis was performed using SPSS 27.0 (data analysis) and GraphPad Prism 9 (graphing). Normally distributed continuous data were analyzed by independent *t*‐test (two independent groups), paired *t*‐test (two paired groups), or repeated‐measures ANOVA (≥ 3 groups); non‐normally distributed data used Mann–Whitney U‐test (independent), Wilcoxon signed‐rank test (paired), or Friedman test (≥ 3 groups). Improvement rate ([T0d‐T28d]/T0d) was calculated only when significant intergroup differences existed (*p* < 0.05).

## 3 Results

This study included a total of 35 participants, with 2 dropouts, resulting in 33 valid data sets. Among the participants, 4 were male, 29 were female, with a mean age of 26.20 ± 7.22 years.

### 3.1 Subjective Assessment

#### 3.1.1 Both the Experimental and Control Cream Improved Subjective Symptoms, With the Experimental Cream Showing Superior Efficacy in Alleviating Pruritus

**FIGURE 1:**
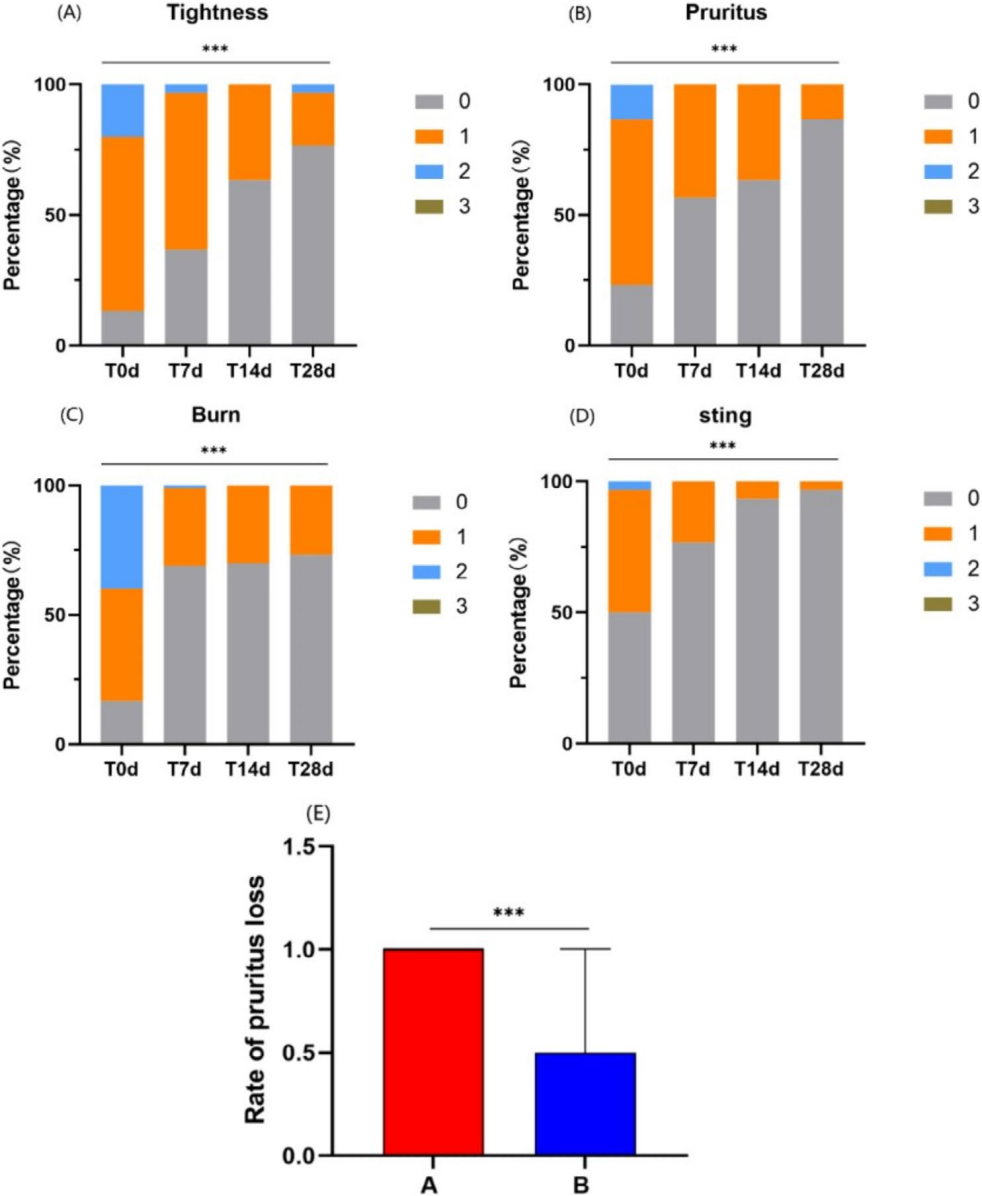
Comparison of the symptom scores of Group A at Days 0, 7, 14, and 28, and the comparison of pruritus improvement rates between Group A and Group B. (A) Tightness score. (B) Pruritus score. (C) Burn score. (D) Sting score. (E) Comparison of pruritus improvement rates between Group A and Group B. (Using Friedman test and U‐test, ***p < 0.001).

After 28 days of using the experimental cream and the control cream:

Group A showed significant reductions in the subjective symptom scores for tightness (Figure 1A), pruritus (Figure 1B), burn (Figure 1C), and sting (Figure 1D) (*p* < 0.001). Group B also showed significant reductions in tightness, pruritus, burn, and sting scores (pruritus score *p* < 0.01, other indices *p* < 0.001).

Group A's pruritus score decreased from 1.00 (0.75, 1.00) to 0.00 (0.00, 0.00) (*p* < 0.001), while Group B's pruritus score decreased from 1.00 (0.75, 1.00) to 1.00 (0.00, 1.00) (*p* < 0.01). The improvement rate in pruritus scores for Group A was higher than that of Group B (using the U‐test, Z = ‐3.774, *p* < 0.001) (Figure 1E). There were no statistically significant differences in the improvement rates of the other indices between Group A and Group B (*p* > 0.05).

#### 3.1.2 Both the Experimental Cream and the Control Cream Improved Objective Signs, With the Experimental Cream Sowing Superior Efficacy in Reducing Erythema

**FIGURE 2:**
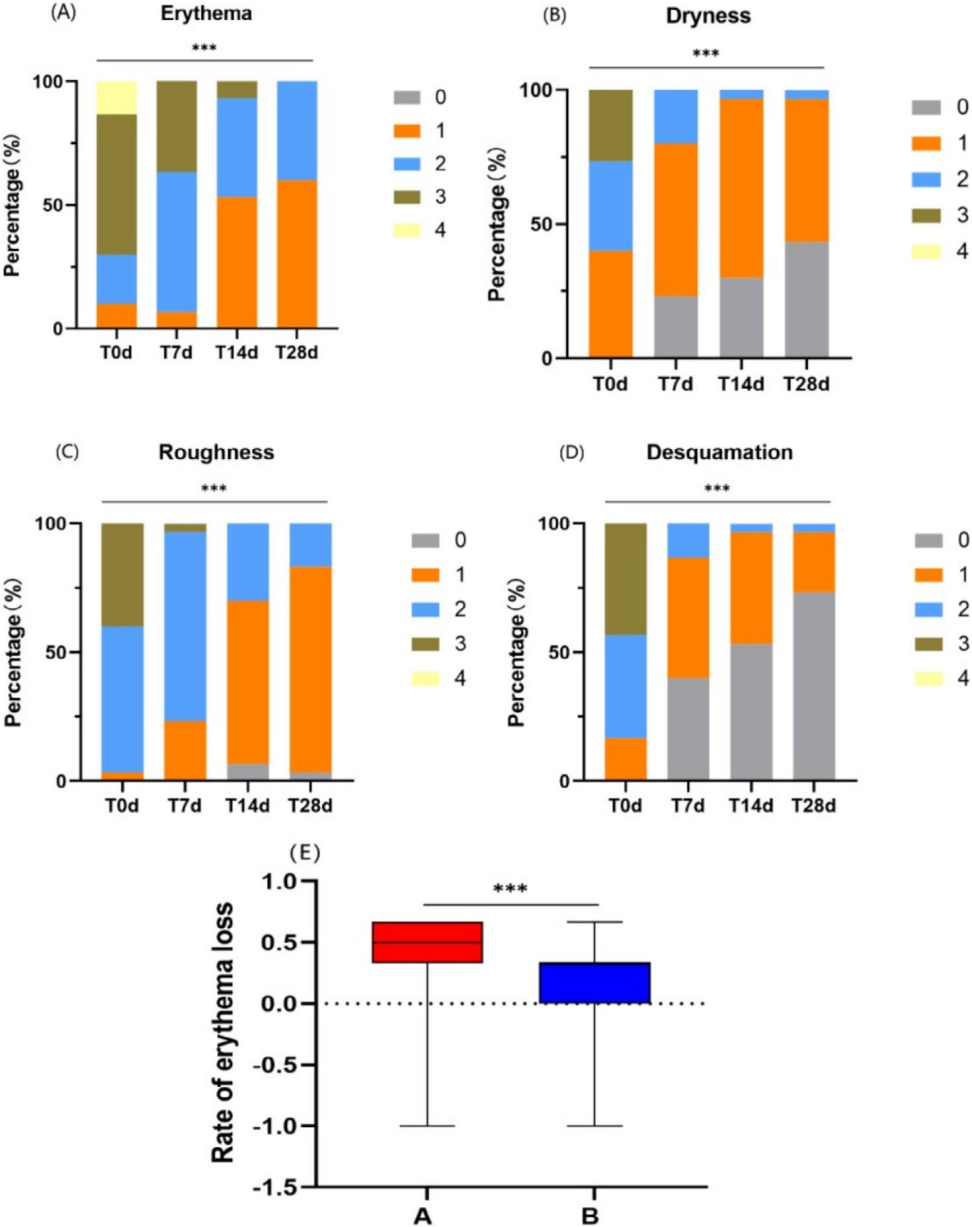
Comparison of clinical scores by doctors for Group A at Days 0, 7, 14, and 28, and comparison of erythema improvement rates between Group A and Group B. (A) Erythema score. (B) Dryness score. (C) Roughness score. (D) Desquamation score. (E) Comparison of erythema improvement rates between Group A and Group B. (Using Friedman test and U‐test, ***p < 0.001).

After 28 days of using the experimental cream and the control cream:

Group A showed significant reductions in erythema (Figure 2A), dryness (Figure 2B), roughness (Figure 2C), and desquamation (Figure 2D) scores (*p* < 0.001). Group B also showed significant reductions in erythema, dryness, roughness, and desquamation scores (*p* < 0.001).

Group A's erythema score decreased from 3.00 (2.00, 3.00) to 1.00 (1.00, 1.00) (*p* < 0.001), while Group B's erythema score decreased from 3.00 (2.00, 3.00) to 2.00 (2.00, 2.00) (*p* < 0.001). The improvement rate in erythema scores for Group A was higher than that of Group B (using the U‐test, Z =−3.400, *p* < 0.001) (Figure 2E). There were no statistically significant differences in the improvement rates of the other indices between Group A and Group B (*p* > 0.05).

### 3.2 Semi‐Subjective Assessment

#### 3.2.1 Both the Experimental Cream and the Control Cream Reduced the LAST Score, With the Experimental Cream Reducing the CAT Score

**FIGURE 3:**
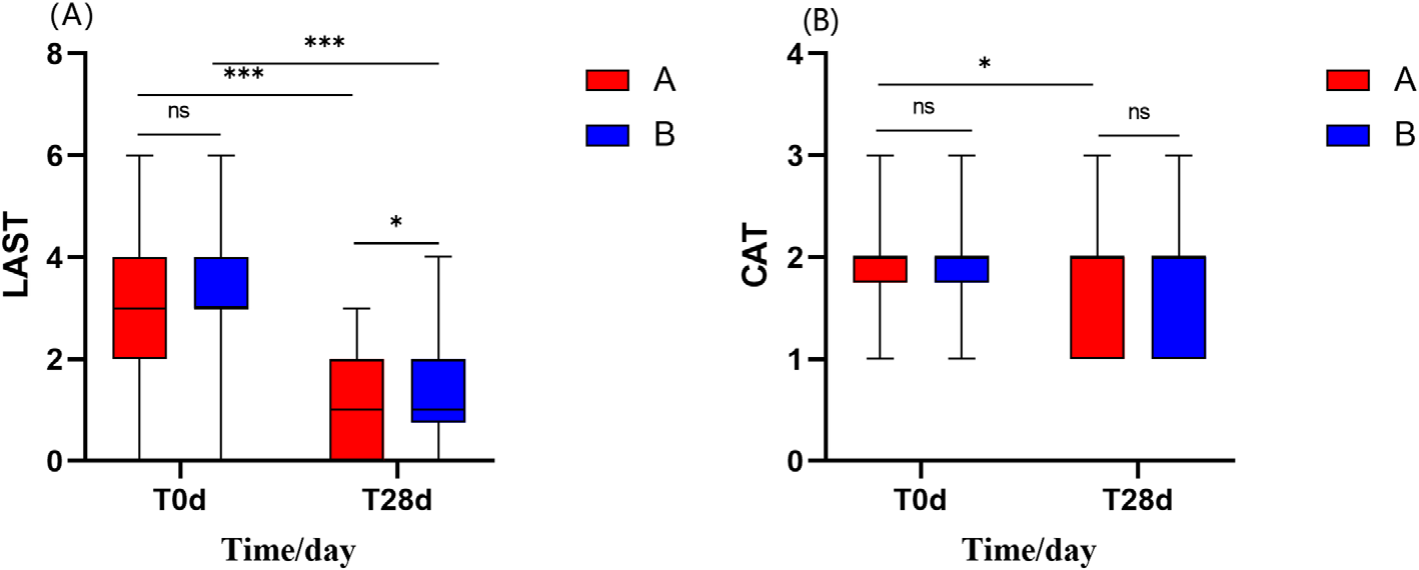
Comparison of LAST and CAT scores between Group A and Group B at Days 0 and 28. (A) LAST score. (B) CAT score. (Using sign‐rank test and U‐test, *p < 0.05, ***p < 0.001).

After 28 days of using the experimental cream and the control cream:

Group A showed a decrease in LAST scores from 3.00 (2.00, 4.00) to 1.00 (0.00, 2.00) (*p* < 0.001), while Group B showed a decrease in LAST scores from 3.00 (3.00, 4.00) to 1.00 (0.75, 2.00) (*p* < 0.001) (Figure 3A). Group A's CAT score decreased from 2.00 (1.75, 2.00) to 2.00 (1.00, 2.00) (*p* < 0.05), while Group B's CAT score decreased from 2.00 (1.75, 2.00) to 2.00 (1.00, 2.00) (*p* > 0.05) (Figure 3B).

The improvement rate in LAST scores between Group A and Group B showed no significant difference (*p* > 0.05). Only Group A showed a significant change in the CAT score before and after treatment, so a comparison of CAT improvement rates between Group A and Group B was not conducted.

### 3.3 Objective Assessment

#### 3.3.1 Both the Experimental Cream and the Control Cream Reduced Facial Eythema, With the Experimental Cream Showing Superior Efficacy in Reducing Erythema

**FIGURE 4:**
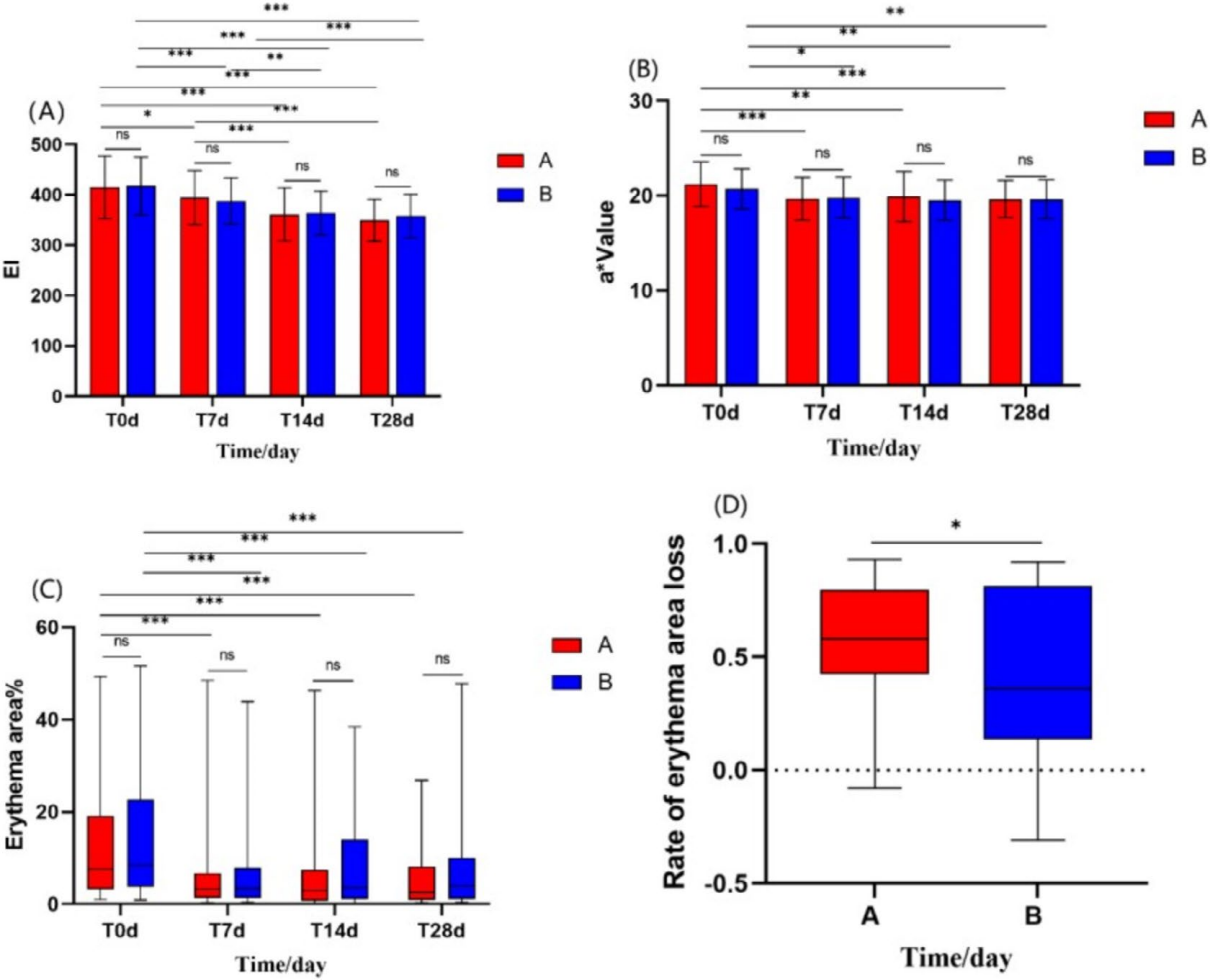
Comparison of facial erythema reduction between Group A and Group B after 28 days. (A) Erythema index (EI). (B) a* Value. (C) Facial erythema area ratio. (D) Comparison of erythema area ratio improvement rates between Group A and Group B. (EI and a* values were analyzed using repeated‐measures ANOVA and independent t‐tests. Erythema area ratio was assessed with Friedman test and Mann–Whitney U‐test (*p < 0.05, **p < 0.01, ***p < 0.001).)

After 28 days of using the experimental cream and the control cream:

Both Group A and Group B showed significant reductions in the erythema index (EI), a* value, and the proportion of facial erythema area (*p* < 0.001). Group A's EI decreased from 414.71 ± 62.17 to 349.10 ± 41.78 (*p* < 0.001), and Group B's EI decreased from 417.64 ± 57.31 to 357.43 ± 43.43 (*p* < 0.001) (Figure 4A); Group A's a* value decreased from 21.21 ± 2.35 to 19.65 ± 1.96 (*p* < 0.001), and Group B's a* value decreased from 20.71 ± 2.14 to 19.62 ± 2.04 (*p* < 0.001) (Figure 4B); Group A's facial erythema area ratio decreased from 7.94 (3.11, 21.35) % to 2.50 (0.91, 8.23) % (*p* < 0.001), while Group B's erythema area ratio decreased from 9.55 (3.71, 28.13) % to 3.72 (1.11, 10.32) % (*p* < 0.001) (Figure 4C).

The improvement rate in erythema area ratio for Group A was higher than that of Group B (using the U‐test, Z =−2.203, *p* < 0.05) (Figure 4D). There was no significant difference between Group A and Group B in the improvement rates of EI and a* value (*p* > 0.05).

**FIGURE 5:**
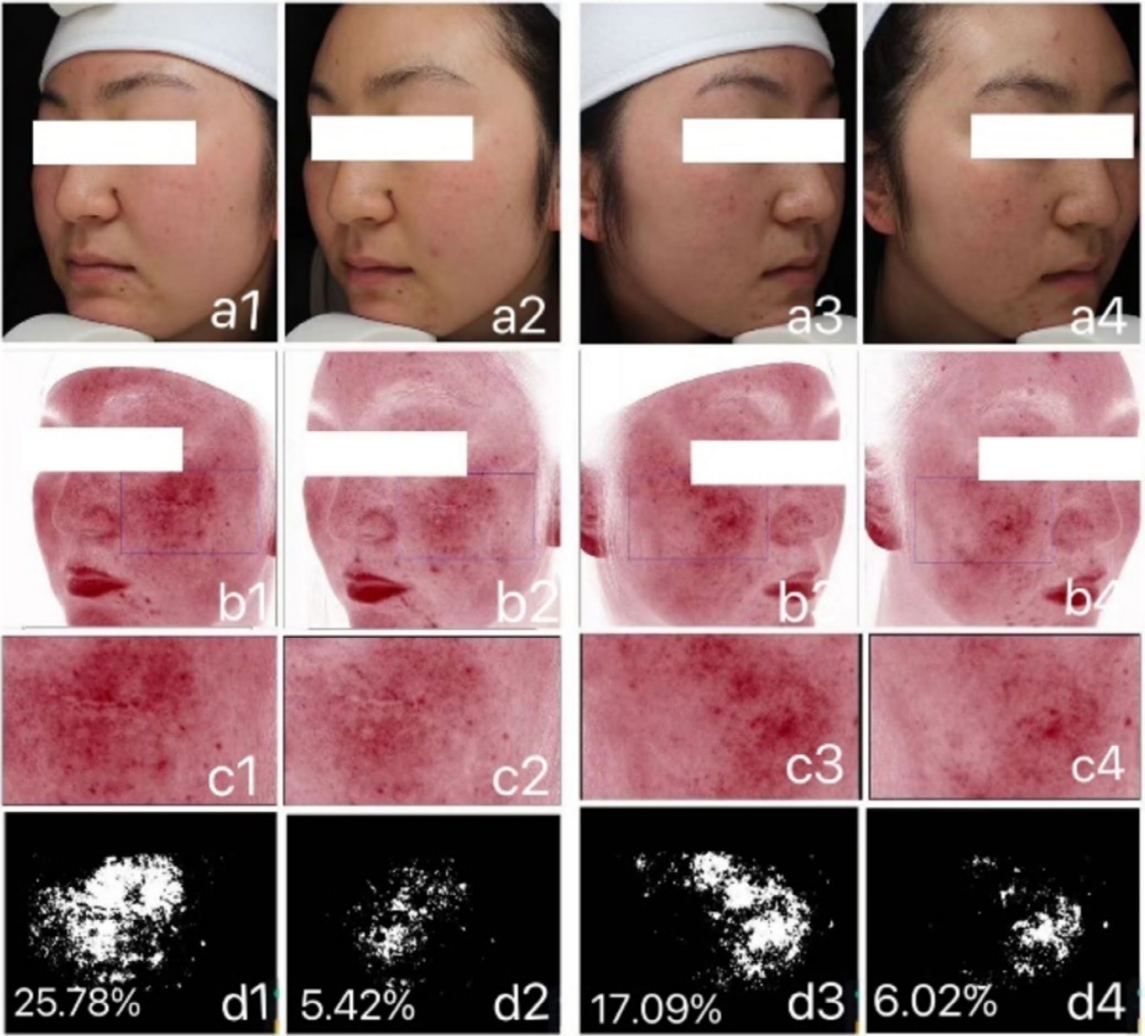
Typical case images of erythema area ratio. (a1–d1) represent the status of Group A at Day 0 (T0d), (a2–d2) represent the status of Group A at Day 28 (T28d); (a3–d3) represent the status of Group B at T0d, (a4–d4) represent the status of Group B at T28d. (a–d) represent: White light photo, red area photo, selected erythema region, and output results.

Representative images of erythema area ratio (Figure 5).

#### 3.3.2 The Experimental Cream Can Reduce Neuro‐Sensitivity

**FIGURE 6:**
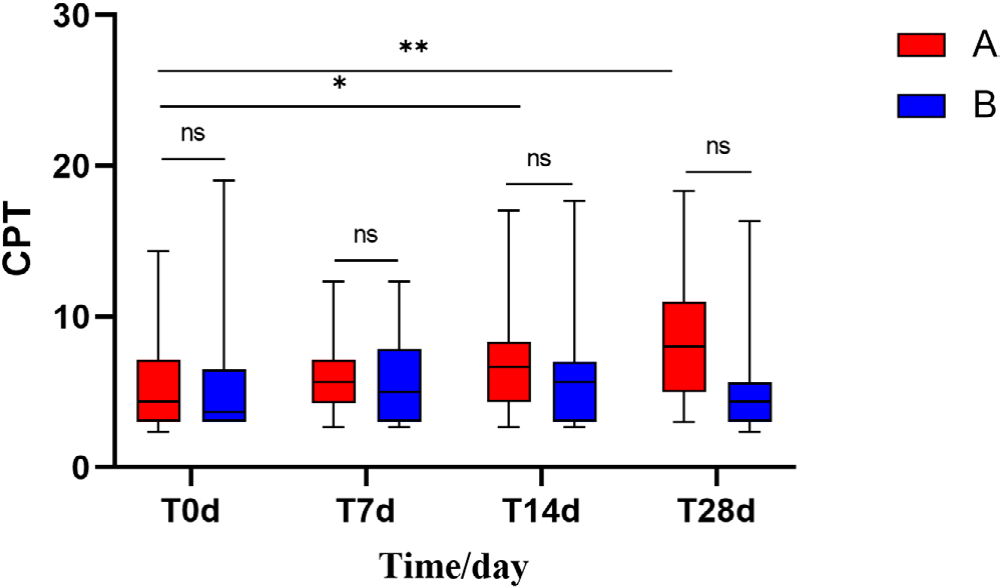
Comparison of CPT values between Group A and Group B at Days 0, 7, 14, and 28 (Using Friedman test and U‐test, *p < 0.05, **p < 0.01).

After 28 days of using the experimental cream and the control cream:

Group A showed a significant increase in CPT values, while Group B showed no significant change in CPT values. Group A's CPT value increased from 4.30 (3.00, 7.20) to 8.00 (5.00, 11.00) (*p* < 0.01), whereas Group B's CPT value increased from 3.70 (3.00, 6.50) to 4.30 (3.00, 5.70) (*p* > 0.05) (Figure 6).

There was a statistical difference in the CPT values before and after treatment in Group A only, so no comparison of the improvement rate of CPT values was made between Group A and Group B.

#### 3.3.3 Both the Experimental Cream and the Control Cream Can Repair the Skin Barrier and Reduce TEWL Values, With the Experimental Cream Showing Superior Efficacy

**FIGURE 7:**
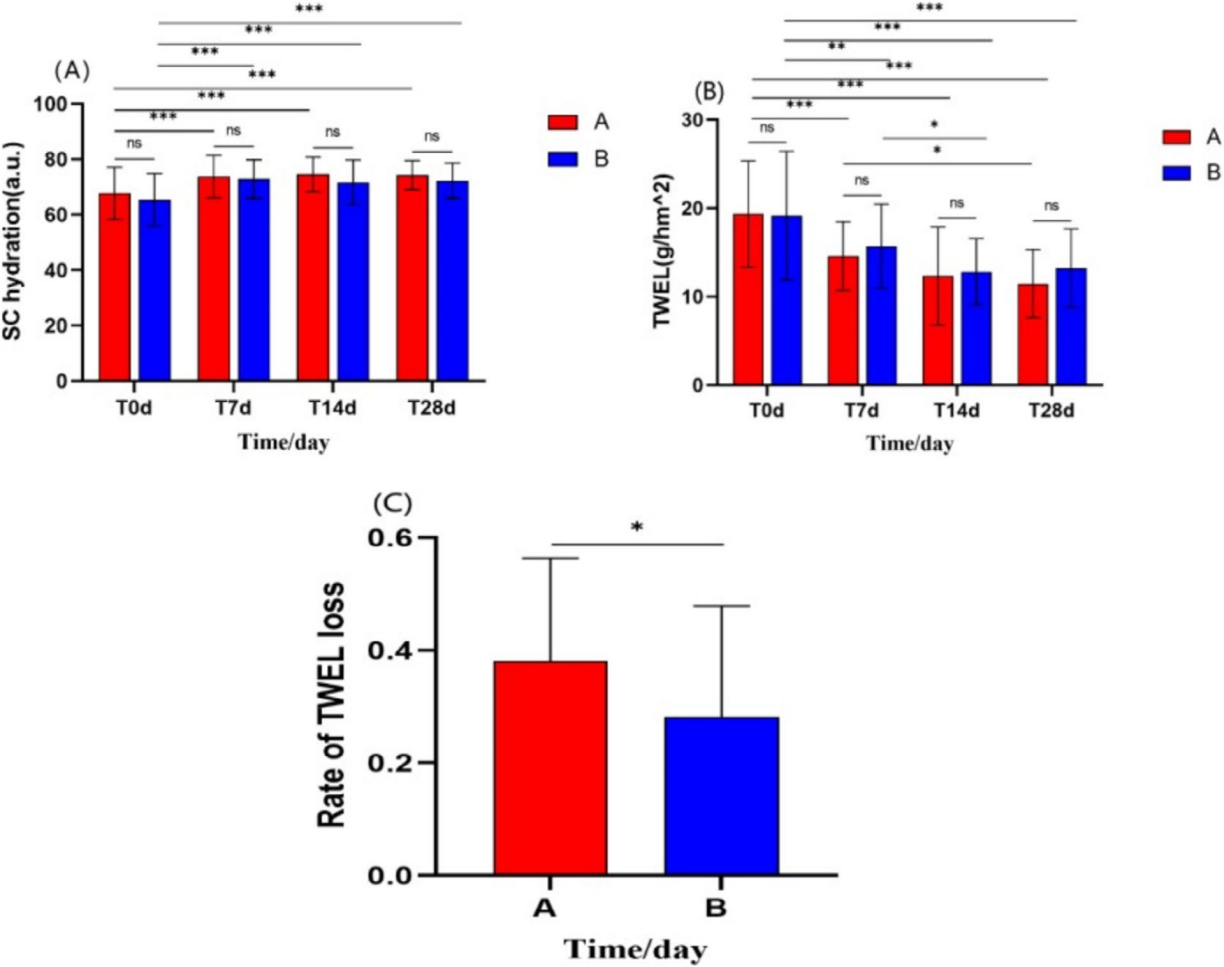
Comparison of SC hydration, TEWL values between Group A and Group B at Days 0, 7, 14, and 28, and comparison of TEWL improvement rates between Group A and Group B. (A) SC hydration. (B) TEWL values. (C) Comparison of TEWL improvement rates between Group A and Group B. (Using repeated‐measures ANOVA and independent samples *t*‐test, *p < 0.05, **p < 0.01, ***p < 0.001).

After 28 days of using the experimental cream and the control cream:

Both Group A and Group B showed significant increases in stratum corneum hydration and significant reductions in TEWL values. Group A's stratum corneum hydration increased from 67.73 ± 9.42 to 74.29 ± 5.23 (*p* < 0.01), while Group B's hydration increased from 65.39 ± 9.43 to 72.23 ± 6.38 (*p* < 0.001) (Figure 7A). Group A's TEWL value decreased from 19.34 ± 6.02 to 11.46 ± 3.84 (*p* < 0.001), and Group B's TEWL value decreased from 19.15 ± 7.28 to 13.22 ± 4.34 (*p* < 0.001) (Figure 7B).

The improvement rate in TEWL values for Group A was higher than that of Group B (using independent samples *t*‐test, *t* = 2.026, *p* < 0.05) (Figure 7C). There was no statistically significant difference between Group A and Group B in the improvement rates of stratum corneum hydration (*p* > 0.05).

#### 3.3.4 The Experimental Cream and the Control Cream Showed No Significant Improvement in Sebum or L* Values

After 28 days of using the experimental cream and the control cream, there were no statistically significant differences in the sebum or L* values between Group A and Group B (*p* > 0.05).

### 3.4 Other

#### 3.4.1 Both the Experimental Cream and the Control Cream Improved the Quality of Life of the Participants

**FIGURE 8:**
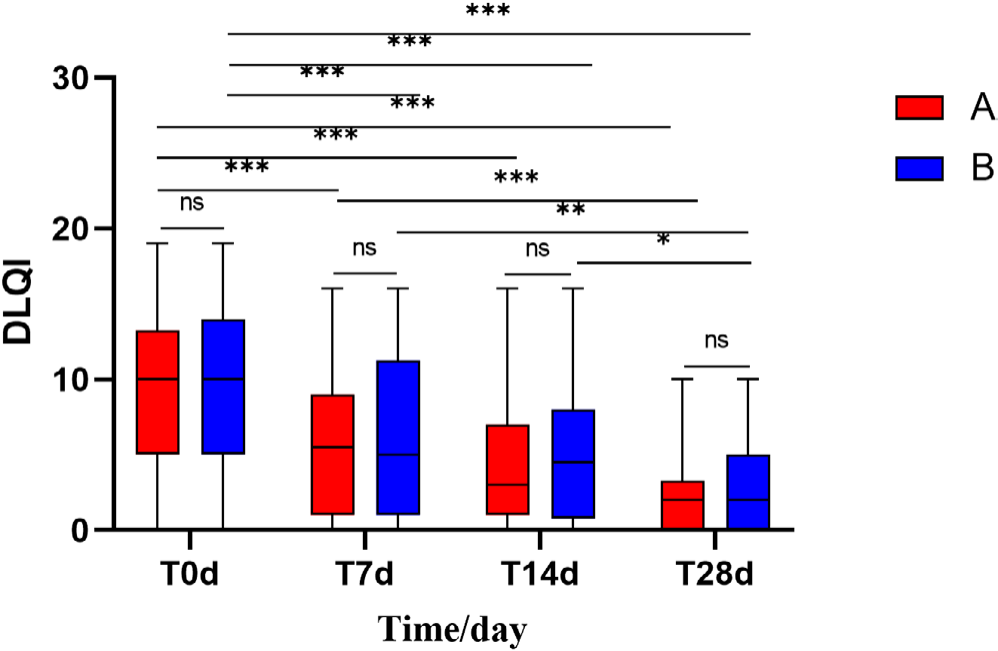
Comparison of DLQI scores between Group A and Group B at Days 0, 7, 14, and 28. (Using Friedman test and U‐test, *p < 0.05, **p < 0.01, ***p < 0.001).

After 28 days of using the experimental cream and the control cream:

Both Group A and Group B showed significant reductions in DLQI scores. Group A's DLQI score decreased from 10.00 (5.00, 13.30) to 2.00 (0.00, 3.00) (*p* < 0.001), and Group B's DLQI score decreased from 10.00 (5.00, 14.00) to 2.00 (0.00, 5.00) (*p* < 0.001) (Figure 8).

There was no significant difference in the DLQI improvement rates between Group A and Group B (*p* > 0.05).

#### 3.4.2 Both the Experimental Cream and the Control Cream Provided a Good User Experience

**FIGURE 9:**
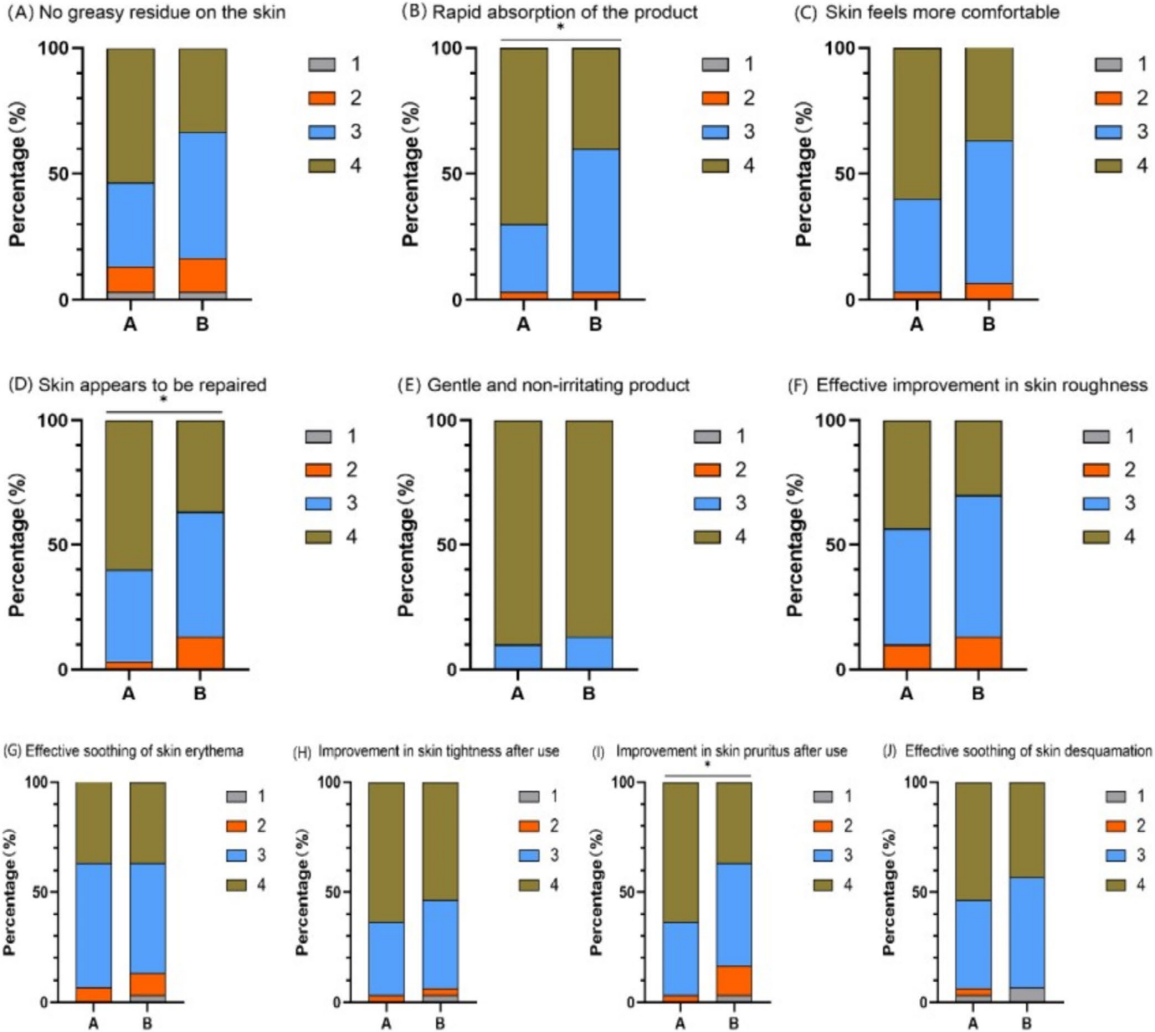
Product user experience ratings for Group A and Group B. (A) No greasy residue on skin. (B) Fast absorption. (C) Skin feels more comfortable. (D) Skin appears repaired. (E) Product is mild and non‐irritating. (F) Product effectively improves skin roughness. (G) Effectively soothes skin redness. (H) Improves skin tightness. (I) Alleviates pruritus. (J) Effectively soothes skin desquamation. (Using U‐test, *p < 0.05).

After 28 days of using the experimental cream and the control cream:

Both the experimental cream and the control cream received good user experience ratings. Over 86.6% of participants in Group A and over 83.3% of participants in Group B agreed or strongly agreed with 8 user experience evaluation items (Figure 9).

Group A showed superior results in terms of fast absorption, improvement in skin appearance, and alleviation of pruritus compared to Group B (*p* < 0.05) (Figure 9B,D,I).

#### 3.4.3 Typical Case Images for the Experimental Cream

**FIGURE 10:**
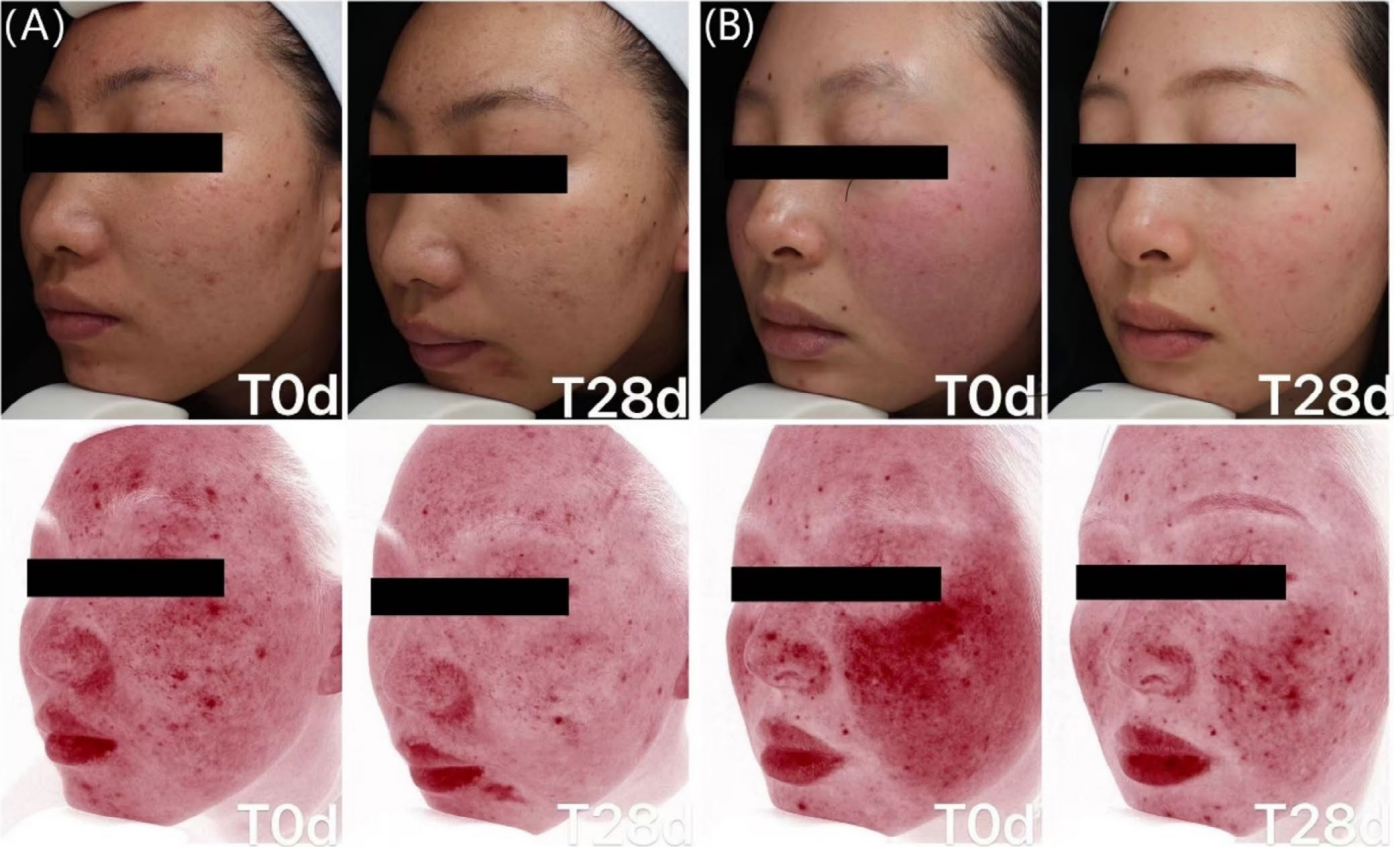
（A) and (B) are typical case images for the experimental cream.

Typical case comparison before and after 28 days of using the experimental cream (Figure 10).

### 3.5 Safety

#### 3.5.1 Both the Experimental Cream and the Control Cream Demonstrated Good Tolerability With no Adverse Reactions

After 28 days of using the experimental cream and the control cream:

Only 6% of participants in Group A experienced mild temporary subjective symptoms within 7 days, and only 3% of participants in Group B experienced mild temporary symptoms within 7 days. At the T14d and T28d follow‐up, neither Group A nor Group B participants showed any subjective or objective symptoms.

No adverse reactions occurred in either Group A or Group B during the study period or the 28‐day safety monitoring period following the study.

## 4 Discussion

The results of this experiment show that both the experimental cream and the control cream can effectively improve the symptoms and signs of sensitive skin, increase the hydration of the stratum corneum, and reduce TEWL values, LAST, and DLQI scores. Meanwhile, the experimental cream demonstrates superior efficacy in alleviating pruritus, erythema, and TEWL, and also offers the unique advantage of lowering CAT scores and increasing CPT values.

International studies have shown that products containing spring water can suppress inflammation, reduce post‐desquamation erythema, and soothe sensitive skin [19]. Products with 4‐t‐butylcyclohexanol [20], acetyldipeptide‐1 cetyl ester [21], and licochalcone A [22] have shown calming effects on sensitive skin. Previous research from our team found that a cream containing purslane extract and prinsepia utilis oil improved SC hydration and skin texture [13]. Another study found that a herbal cream containing Angelica polymorpha sinensis root extract and *porphyridium cruentum* increased SC hydration, reduced TEWL, and significantly alleviated facial erythema [23]. The above studies mainly focus on skin barrier function and erythema in sensitive skin, but relatively little attention has been paid to the symptoms and neurovascular hyper‐reactivity of sensitive skin. Moreover, these studies have not used objective indicators to quantitatively analyze skin neuro‐sensitivity.

In the current study, the experimental cream was developed based on the formula of the Winona Sensitive Moisturizing Special Care Cream (First Generation) and incorporated four active ingredients: prinsepia utilis Royle polysaccharides, *physalis alkekengi* calyx extract, *salvia miltiorrhiza* root extract, and *porphyridium cruentum* extract. CPT values were used to assess neuro‐sensitivity. The results showed that the experimental cream not only increased the hydration of the stratum corneum, reduced TEWL values, and lowered LAST and DLQI scores, but also improved sensitive skin symptoms, signs, CPT values, and CAT scores. This may be related to the fact that the ingredients of the cream not only repair the skin barrier, exhibit anti‐inflammatory, moisturizing, and angiogenesis‐inhibiting properties, but also reduce neurovascular hyper‐reactivity.

The various active ingredients in this moisturizer target different mechanisms involved in sensitive skin, improving skin sensitivity. Prinsepia utilis is rich in lipids, which are not only used for food and medicinal purposes but are also widely utilized in functional cosmetics. Prinsepia utilis oil not only has analgesic and anti‐inflammatory effects [24], but also promotes the synthesis of ceramide in keratinocytes and repairs the damaged epidermal permeability barrier [25]. Some researchers have extracted a valuable active substance, prinsepia utilis Royle polysaccharides, from the seed pomace. Prinsepia utilis Royle polysaccharides inhibit the content of substance P and act directly on the CLDN5 gene to enhance intercellular tight junctions, thereby strengthening the skin barrier, improving skin moisture retention, resistance, and epidermal thickness, and reducing permeability.

The experimental cream showed superior efficacy in alleviating pruritus, facial erythema, and TEWL values compared to the control cream, and its unique advantages in increasing CPT values and lowering CAT scores may be related to the *Silybum marianum* polysaccharide in the cream [14].

*Salvia miltiorrhiza* root extract inhibits neurogenic pain by suppressing microglial activation and immune responses [26]. It also reduces the expression of TRPV1 and lowers the levels of inflammatory cytokines IL‐1β, IL‐6, and TNF‐α [16]. The experimental cream alleviates symptoms such as pruritus, burning, pain, and erythema, increases CPT values, and lowers CAT scores, possibly due to the effect of *salvia miltiorrhiza* root extract in reducing TRPV1 expression. *Physalis alkekengi* calyx extract contains various compounds, such as flavonoids, alkaloids, polysaccharides, and piperazine derivatives, with its primary pharmacological action being anti‐inflammatory [17]. The experimental cream showed superior efficacy in reducing erythema scores and erythema area, likely due to the anti‐inflammatory properties of *physalis alkekengi* calyx extract. *Porphyridium cruentum* has been shown to enhance vascular elasticity, possess antioxidant and moisturizing effects [15], and alleviate inflammation [27, 28]. The experimental cream showed superior efficacy in reducing erythema scores and erythema area compared to the control cream, likely due to the vascular elasticity‐enhancing and anti‐inflammatory effects of *porphyridium cruentum* . Studies suggest that *Portulaca oleracea* has anti‐inflammatory properties [29], as well as antioxidant, anti‐ulcer, and anticancer activities [30]. β‐glucan accelerates skin wound healing [31], repairs the skin barrier, and provides moisturizing benefits [32]. Sodium hyaluronate, a natural moisturizing factor, exerts anti‐inflammatory effects, improves barrier function, and enhances stratum corneum (SC) hydration [33].

Therefore, after adding new active ingredients, the experimental product, based on the efficacy of the control product, not only suppresses inflammation from multiple dimensions and enhances barrier repair but also reduces neurovascular hypersensitivity through different mechanisms, providing a new option for those troubled by sensitive skin. However, this study also has limitations. Since multiple tests are required to obtain CPT values, participants may develop tolerance to the stimulation, which may sometimes affect the stability of the results. Future improvements may involve combining methods like the BoSS questionnaire.

## 5 Conclusions

This study indicates that the novel cream demonstrates good safety. Both the novel cream and Winona Sensitive Moisturizing Special Care Cream are effective in improving the symptoms and signs of sensitive skin, as well as in repairing the skin barrier. Moreover, the novel cream performs better than the Winona Sensitive Moisturizing Special Care Cream in improving pruritus, erythema, and skin barrier function, and it also offers the unique advantage of reducing neurovascular hyper‐reactivity.

## Author Contributions

L.J., S.Y., and M.L. performed the research. L.J. and J.O. designed the research study. L.H. contributed essential reagents or tools. L.J., D.W., and Z.X. analyzed the data. L.J. wrote the paper. All authors have read and approved the final manuscript.

## Conflicts of Interest

The authors declare no conflicts of interest.

## Data Availability

The authors have nothing to report.
